# Fungal Chitin Dampens Inflammation through IL-10 Induction Mediated by NOD2 and TLR9 Activation

**DOI:** 10.1371/journal.ppat.1004050

**Published:** 2014-04-10

**Authors:** Jeanette Wagener, R. K. Subbarao Malireddi, Megan D. Lenardon, Martin Köberle, Simon Vautier, Donna M. MacCallum, Tilo Biedermann, Martin Schaller, Mihai G. Netea, Thirumala-Devi Kanneganti, Gordon D. Brown, Alistair J. P. Brown, Neil A. R. Gow

**Affiliations:** 1 Aberdeen Fungal Group, School of Medical Sciences, Institute of Medical Sciences, University of Aberdeen, Aberdeen, United Kingdom; 2 Department of Immunology, St. Jude Children's Research Hospital, Memphis, Tennessee, United States of America; 3 Department of Dermatology, Eberhard Karls University Tübingen, Tübingen, Germany; 4 Department of Internal Medicine and Nijmegen Institute for Infection, Inflammation & Immunity (N4i), Radboud University Nijmegen Medical Center, Nijmegen, The Netherlands; University of California, San Francisco, United States of America

## Abstract

Chitin is an essential structural polysaccharide of fungal pathogens and parasites, but its role in human immune responses remains largely unknown. It is the second most abundant polysaccharide in nature after cellulose and its derivatives today are widely used for medical and industrial purposes. We analysed the immunological properties of purified chitin particles derived from the opportunistic human fungal pathogen *Candida albicans*, which led to the selective secretion of the anti-inflammatory cytokine IL-10. We identified NOD2, TLR9 and the mannose receptor as essential fungal chitin-recognition receptors for the induction of this response. Chitin reduced LPS-induced inflammation *in vivo* and may therefore contribute to the resolution of the immune response once the pathogen has been defeated. Fungal chitin also induced eosinophilia *in vivo*, underpinning its ability to induce asthma. Polymorphisms in the identified chitin receptors, NOD2 and TLR9, predispose individuals to inflammatory conditions and dysregulated expression of chitinases and chitinase-like binding proteins, whose activity is essential to generate IL-10-inducing fungal chitin particles *in vitro*, have also been linked to inflammatory conditions and asthma. Chitin recognition is therefore critical for immune homeostasis and is likely to have a significant role in infectious and allergic disease.

**Authors Summary:**

Chitin is the second most abundant polysaccharide in nature after cellulose and an essential component of the cell wall of all fungal pathogens. The discovery of human chitinases and chitinase-like binding proteins indicates that fungal chitin is recognised by cells of the human immune system, shaping the immune response towards the invading pathogen. We show that three immune cell receptors– the mannose receptor, NOD2 and TLR9 recognise chitin and act together to mediate an anti-inflammatory response via secretion of the cytokine IL-10. This mechanism may prevent inflammation-based damage during fungal infection and restore immune balance after an infection has been cleared. By increasing the chitin content in the cell wall pathogenic fungi may influence the immune system in their favour, by down-regulating protective inflammatory immune responses. Furthermore, gene mutations and dysregulated enzyme activity in the described chitin recognition pathway are implicated in inflammatory conditions such as Crohn's Disease and asthma, highlighting the importance of the discovered mechanism in human health.

## Introduction

Chitin is a robust β-1,4-linked homopolymer of *N*-acetylglucosamine (GlcNAc) and is an essential polysaccharide of the cell wall of all fungal pathogens. It is absent in humans but is also found in the skeletal elements in oomycetes, insects, crustaceans and parasitic nematodes [1], [2], [3], [4]. In terms of global biomass, chitin is the second most abundant polysaccharide in nature after cellulose.

*Candida albicans* is a common mucosal pathogen in humans that can cause life-threatening infections in patients suffering trauma or immune dysfunction [5]. Under normal growth conditions, chitin is a minor component of the *C. albicans* cell wall, comprising only 2 to 3% of its dry weight [6]. However, the chitin content of the fungal cell wall increases in response to β-1,3 glucan damage, for example as inflicted by echinocandin antifungal drug treatment [7]. This leads to chitin exposure at the cell surface as well as reduced efficacy of caspofungin *in vitro* and *in vivo* [8]. The discovery of human chitinases and chitinase-like proteins (CLPs), including some that are constitutively expressed by macrophages and epithelial cells of the lung and digestive tract, suggests the presence of a first line of defence against chitin-containing pathogens, as well as mechanisms for chitin recognition, breakdown and immune-modulation in the human host [9], [10]. Dysregulated chitinase- and CLP-expression has been linked to inflammatory and allergic conditions such as asthma and inflammatory bowel disease [11]. Furthermore, recent work has identified additional chitin binding proteins, such as RegIIIg (HIP/PAP), a C-type lectin secreted by Paneth cells in the small intestine that also binds peptidoglycan from Gram-positive bacteria [12], and FIBCD1, a high affinity receptor for chitin and chitin fragments that is highly expressed in the gastrointestinal tract [9].

Studies performed using commercially available sources of chitin from crab/shrimp shell, suggest that the ability of chitin to modulate immune responses depends on the size of the chitin particle. Medium-sized chitin particles (40–70 µm) have been shown to activate TNF and IL-17 production in a TLR2- and dectin-1-dependent fashion, whereas small-size chitin particles (<40 µm) are strong stimulators of TNF and IL-10 in a manner that involves TLR2, dectin-1 and the mannose receptor (MR) [13]. TLR2, dectin-1 and NOD2 have also been implicated in the induction of chitotriosidase expression if stimulated by their specific ligands [14]. However, these studies were performed using commercially available sources of chitin from crab/shrimp shell- a source that is likely to be found in marine and shell fish processing environments and is often relatively impure. In terrestrial environments, arthropods and fungi represent the major source of chitin. Such organisms are also common indoor environments, resulting in chronic exposure due to the inhalation of chitin-containing particles.

We set out to identify chitin pattern recognition receptors (PRRs) in myeloid cells and to investigate the relationship between chitin particle size and chitin recognition. We report here that ultra-pure fungal chitin dominantly induces the anti-inflammatory cytokine IL-10 through uptake mediated by the mannose receptor and signalling that involves NOD2 and TLR9 activation. Polymorphisms in these receptors predispose individuals to inflammatory conditions such as asthma or Crohn's disease (CD), conditions that have been shown to be significantly influenced by exposure to chitin. These findings therefore open new avenues for the understanding and treatment of these diseases.

## Results

### Concentration- dependent immune response to chitin

**Figure 1:**
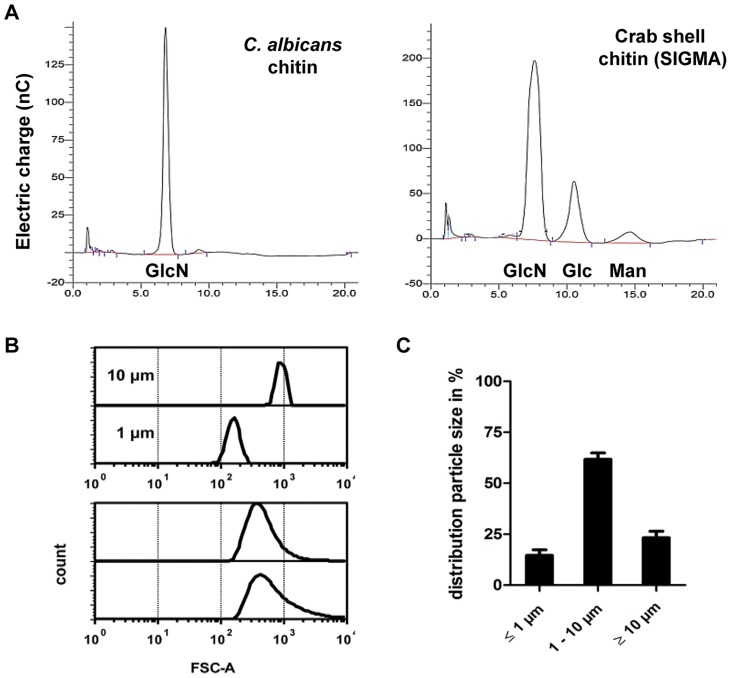
Chitin purity and size. (A) HPLC analysis of TFAA hydrolysed *C. albicans*-chitin (*left*) compared to commercial crab shell chitin (*right*). *GlcN* Glucosamine, *Glc* Glucose, *Man* Mannan. (B) Chitin particle size determined by flow cytometry. (C) Chitin size distribution in percentage of analysed chitin extractions, presented as mean ± SEM, n = 6.

Most immunological studies of chitin have focussed on semi-purified crustacean sources of chitin of relatively large particle size [13]. Here we established the role of ultra-pure fungal cell wall chitin of a determined size in immune regulation. All fungal chitin preparations used for experiments were pyrogen-free, microbiologically sterile and had a purity of over 98% as analysed by HPLC (Fig. 1A). In contrast, commercial crab shell chitin contained only 60% glucosamine (Fig. 1A). All chitin preparations used were between 55% and 100% acetylated, depending on the source of chitin as listed in Table 1. If not stated otherwise, work was performed with chitin extracted from yeast cells of the human pathogen *C. albicans*, which was >97% acetylated. To investigate the size of fungal chitin particles used for experiments, we performed flow cytometry with 1 and 10 µm beads as size standards (Fig. 1B). The majority of fungal derived chitin particles were found to be between 1 to 10 µm in size (Fig. 1C).

**Table 1:** Acetylation degree of purified chitin.

Species	Degree of Acetylation in %
*Candida albicans*	∼97
*Aspergillus fumigatus*	∼93
*Saccharomyces cerevisiae*	∼100
*Mucor circinelloides*	∼95
*Cryptococcus neoformans*	∼55
Crab shell (SIGMA)	∼87

**Figure 2:**
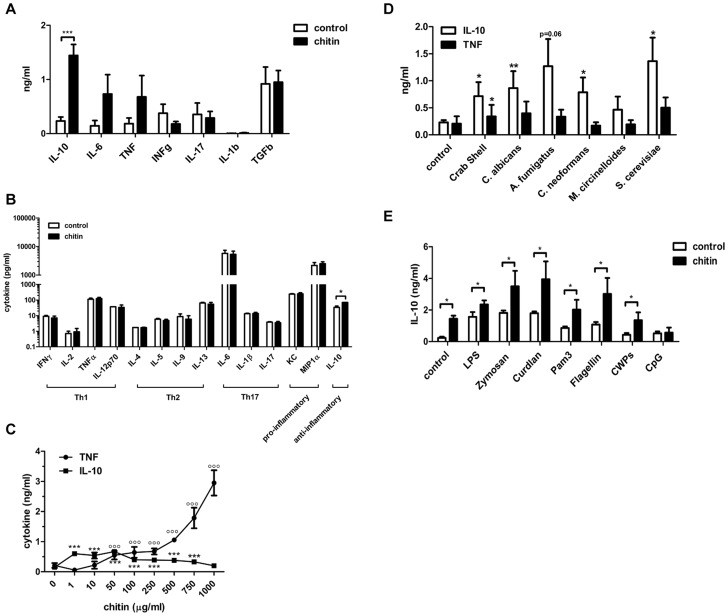
Chitin induced cytokines and synergistic effect on IL-10 secretion. (A) Cytokine induction in hPBMCs incubated with chitin for 24 h, n = 6, ***p<0.001. (B) Cytokine induction in mBMMφ's incubated with chitin for 24 h, n = 4, *p<0.05. (C) IL-10 and TNF induction after 24 h in mBMMφs incubated with increasing chitin concentrations, n = 4, ***p<0.001 for IL-10, °°°p<0.001 for TNF compared to untreated control. (D) IL-10 and TNF induction in hPBMCs incubated with chitin isolated from different species, n = 3, *p<0.05, **p<0.01. (E) Co-incubation of hPBMCs with either LPS, zymosan, curdlan, Pam_3_CSK_4_, flagellin, CpG ODN or *C. albicans* cell wall proteins (CWPs) and chitin, n = 6, *p<0.05. All data are presented as mean values ± SEM.

To characterise the immunological properties of chitin we first analysed a series of cytokines and chemokines in the supernatants of chitin (10 µg/ml) stimulated human peripheral blood mononuclear cells (hPBMCs, 5×10^5^ cells) and mouse bone-marrow derived macrophages (mBMMφs, 1×10^5^ cells). Chitin stimulation significantly increased the secretion of the anti-inflammatory cytokine IL-10 along with the pro-inflammatory cytokines IL-6 and TNF in hPBMCs (Fig. 2A), whereas in mouse macrophages only IL-10 secretion was significantly increased (Fig. 2B). INFγ and IL-17, known to be involved in establishing a protective T-helper (Th) 1/Th17 response to *C. albicans* infections, were not induced in hPBMCs by chitin exposure (Fig. 2A) and chitin did not induce Th1, Th2 or Th17 cytokines in mouse macrophages or any of the pro-inflammatory cytokines/chemokines tested (Fig. 2B).

We tested whether the observed IL-10 induction by fungal chitin was concentration-dependent. Low chitin concentrations (1–10 µg/ml) induced high IL-10 secretion; high chitin concentrations (250–1000 µg/ml) instead strongly induced TNF secretion (Fig. 2C) and intermediate concentrations of chitin (50–100 µg/ml) induced equal amounts of both cytokines (Fig. 2C).

Next we investigated the immunostimulatory effects of chitin particles (10 µg/ml) derived from different fungal species in comparison to purified commercial crab shell chitin. Nearly all tested chitin preparations significantly induced IL-10 secretion in hPBMCs (Fig. 2D) and no obvious differences were observed between different sources of chitin. We also investigated the ability of chitin to block or increase cytokine secretion induced by specific PRR ligands. Co-incubation of hPBMCs (5×10^5^ cells) with lipopolysaccharide (LPS;10 µg/ml), zymosan (10 µg/ml), curdlan (100 µg/ml), Pam_3_CSK_4_ (1 µg/ml), flagellin (100 ng/ml), CpG oligodeoxynucleotides (CpG ODN; 1 µM) or *C. albicans*-derived cell wall mannoproteins (CWP; 100 µg/ml) together with chitin (10 µg/ml) for 24 h resulted in increased IL-10 secretion (Fig. 2E). This increase in IL-10 was significant for all pathogen-associated molecular patterns (PAMPs) tested except for CpG ODN (Fig. 2E), indicating a competitive effect of chitin on CpG-TLR9 interactions. All other cytokines analysed (IL-1β, IL-6, TNF, IFN-γ and TGF-β) showed no significant differences in cytokine induction, although a trend for INF-γ, TGF-β and IL-1β secretion was observed that was ameliorated when hPBMCs were co-stimulated with chitin (Supplementary Fig. S1).

### Mannose receptor-dependent IL-10 secretion

**Figure 3:**
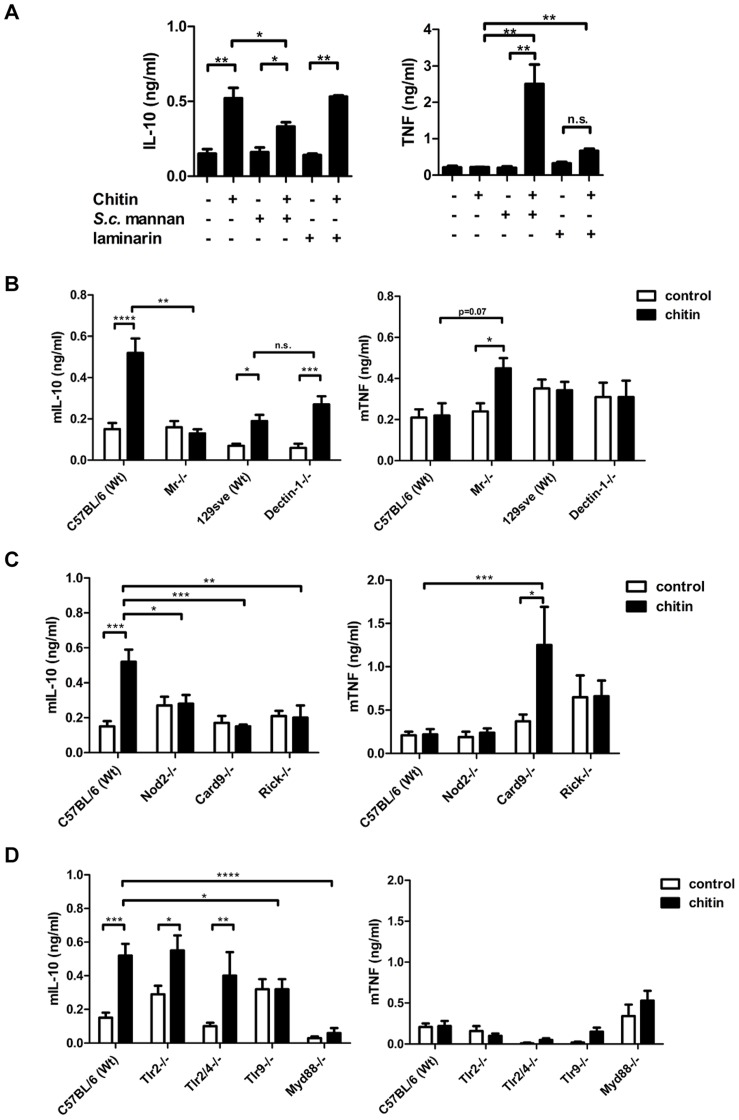
Chitin induced IL-10 secretion depends on mannose receptor, TLR9- and NOD2-signalling. (A) mBMMφs were incubated with *S. c.* mannan or laminarin 1 h prior stimulation with chitin, n = 4. (B) mBMMφs from wild type mice (C57BL/6 and 129Sve), dectin-1- and MR –deficient mice were stimulated with chitin, n = 4. (C) mBMMφs from wild type mice (C57BL/6), NOD2-, RICK- and CARD9-deficient mice stimulated with chitin for 24 h, n = 4. (D) mBMMφs from wild type mice (C57BL/6), TLR2-, TLR2/4-, TLR9- and MyD88-deficient mice stimulated with chitin for 24 h, n = 4. All data are presented as mean values ± SEM, *p<0.05, **p<0.01, ***p<0.001, ****p<0.0001.

We next aimed to identify the receptors whose interaction with fungal chitin mediated the observed IL-10 induction. As mentioned above, intermediate-sized chitin particles (40–70 µm) have been reported to induce a pro-inflammatory immune response (TNF secretion) through dectin-1/TLR2 signalling, whilst small-sized particles (<40 µm) have been reported to induce an anti-inflammatory response (IL-10 secretion) through dectin-1/TLR2 and the mannose receptor [13]. To examine possible chitin-MR/dectin-1 interactions further, we used soluble mannans purified from *Saccharomyces cerevisiae* (*S. c.* mannan) and laminarin (β-1,3 glucan from an alga) to block potential receptor interactions. *S. c.* mannan and laminarin (100 µg/ml) alone were immunological inert and no cytokine response was observed (Fig. 3A). Pre-incubation with *S. c.* mannan reduced chitin-triggered mBMMφ IL-10 secretion by 40%, whilst laminarin treatment did not affect IL-10 secretion (Fig. 3A). Interestingly, blocking MR with *S. c.* mannan significantly increased TNF secretion (Fig. 3A). To confirm these blocking experiments, we exposed mBMMφs from MR- and dectin-1-deficient mice to fungal chitin. Cytokine analysis reinforced the results of the blocking experiments, with increased TNF induction and reduced IL-10 secretion occurring in chitin-stimulated MR-deficient macrophages (Fig. 3B). Dectin-1 deficiency did not influence chitin-triggered IL-10 secretion (Fig. 3B). Blocking dectin-1 in MR-deficient macrophages with laminarin instead confirmed that the observed chitin triggered TNF secretion in *S. c.* mannan pre-treated wild type macrophages (Fig. 3A) and MR-deficient macrophages (Fig. 3B) were Dectin-1 dependent (Supplementary Fig. S2).

### NOD2 and TLR9 mediate IL-10 induction

Chitin is a homopolymer of *N*-acetyl-glucosamine. Together with *N*-acetyl-muramic acid, *N*-acetyl-glucosamine also forms the backbone of bacterial peptidoglycan. Peptidoglycan is known to be recognised by TLR2 and muramyl dipeptide (MDP) by NOD2. Therefore we tested whether chitin is recognised by TLR2 or NOD2 using mBMMφs from TLR2- and NOD2-deficient mice along with mBMMφs from other PRR- and signalling-deficient mutant mice, including TLR2/4-, TLR9-, MyD88-, RICK- and CARD9-knock-outs (Fig. 3C and D). IL-10 secretion was reduced in *Tlr2/4^−/−^* -macrophages, but still significantly increased compared to untreated control cells (Fig. 3D). Similar IL-10 levels were secreted by *Tlr2^−/−^* -macrophages and wild type macrophages. Chitin failed to induce IL-10 secretion in *Nod2^−/−^*-, *Myd88* ^−/−^-, *Card9^−/−^*-, *Rick^−/−^*- and *Tlr9^−/−^* - macrophages over background levels measured in untreated control cells (Fig. 3C and D). Because the IL-10 induction was TLR9-dependent, we also treated the chitin preparations with DNase I prior to stimulation to exclude contaminating DNA as a trigger for IL-10 secretion. DNase I-treated chitin induced similar amounts of IL-10 secretion in wild type macrophages as untreated chitin (Supplementary Fig. S3), indicating a direct role for TLR9 in chitin recognition and induction of IL-10 secretion. This finding is supported by the observed competitive effect of CpG ODN and chitin in hPBMCs (Fig. 2E). Therefore, the intracellular receptors NOD2 and TLR9 together with their downstream signalling adapter proteins are necessary for chitin-induced IL-10 secretion.

### Uptake via MR directs chitin recognition

**Figure 4:**
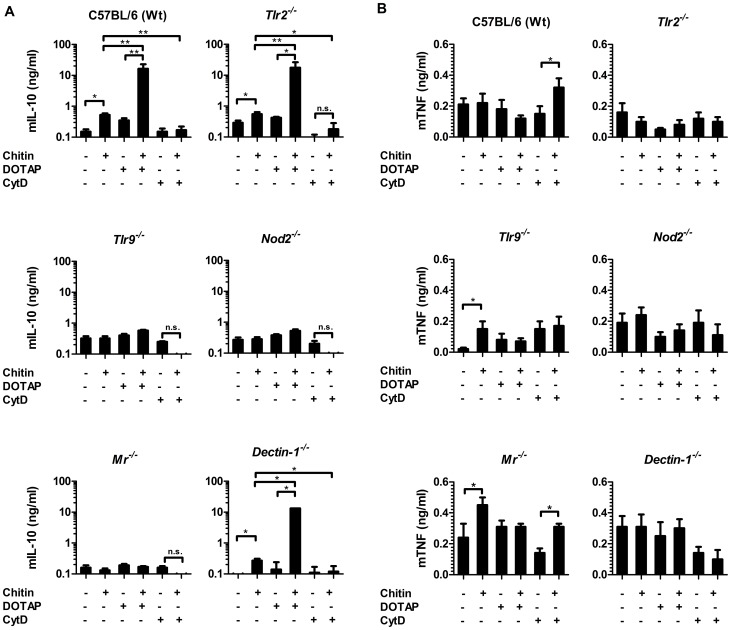
Chitin induced IL-10 secretion requires mannose receptor interaction but not uptake. (A) Chitin was incubated with liposomal transfection reagent DOTAP for 15 min at room-temperature before used to stimulate mBMMφs from wild type, TLR2, TLR9, NOD2, MR and dectin-1-deficient mice for 24 h or (A and B) mBMMφs were treated with CytD for 1 h prior chitin stimulation. Values represent mean ± SEM, n = 4, *p<0.05, **p<0.01.

To further investigate the role of the MR and dectin-1 in chitin recognition, intracellular delivery and induction of IL-10 secretion, we forced chitin uptake into the cytoplasm of wild type, *Tlr2^−/−^*-, *Tlr9^−/−^*-, *Nod2^−/−^*-, *Mr^−/−^*- and *dectin-1^−/−^*-macrophages by liposomal transfection using DOTAP (10 µg/ml), or blocked chitin uptake with the endocytosis inhibitor cytochalasin D (CytD; 5 µM) (Fig. 4). Chitin transfection into wild type, *Tlr2^−/−^*- and *dectin-1^−/−^*- macrophages significantly increased IL-10 secretion, whereas no IL-10 induction occurred in chitin-transfected *Nod2^−/−^*-, *Mr^−/−^*- or *Tlr9^−/−^*- macrophages (Fig. 4A). Moreover, the inhibition of endocytosis with CytD abrogated the IL-10 secretion in all macrophages (Fig. 4A), whereas TNF secretion was significantly increased in wild type and *Mr^−/−^*- macrophages (Fig. 4B). Increased TNF levels were absent in *Tlr2^−/−^*- and *dectin-1^−/−^*- macrophages (Fig. 4B), therefore the observed TNF secretion after inhibition of endocytosis was mediated by TLR2 and dectin-1 signalling.

**Figure 5:**
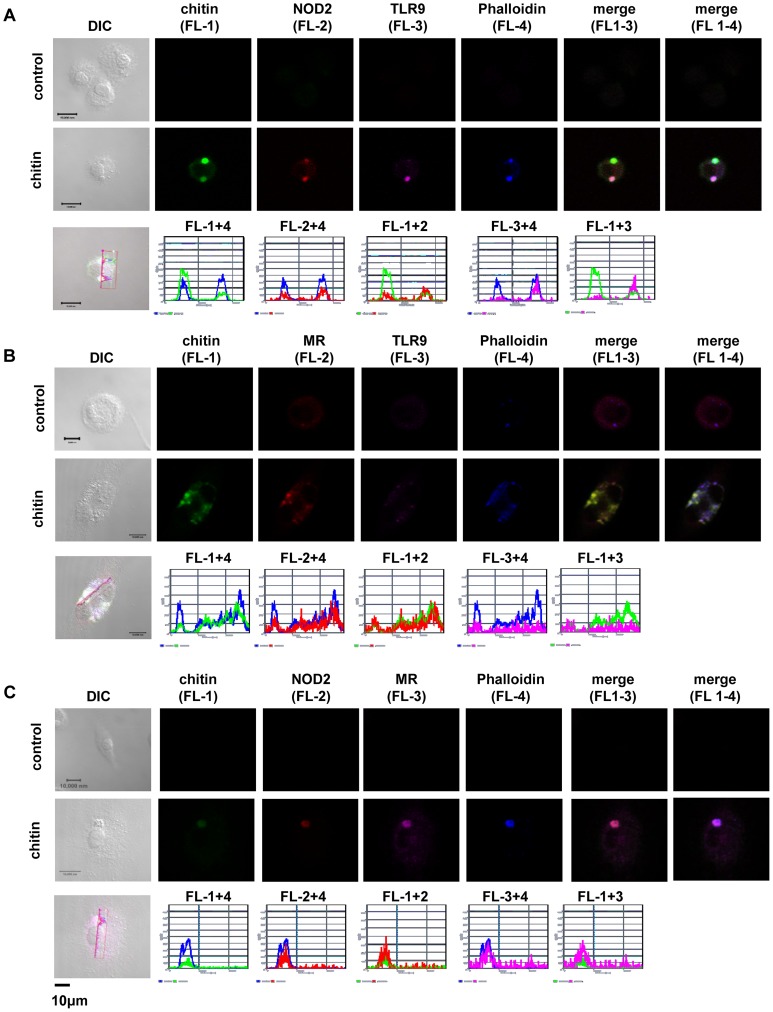
Chitin co-localisation with NOD2, TLR9 and MR. Confocal fluorescence microscopy of mBMMφs from wild type mice stimulated with chitin for 20 min. NOD2 protein was detected with anti-mouse NOD2 antibody, TLR9 with anti-mouse TLR9 antibody, MR with anti-mouse CD206-antibody and chitin was detected using a chitin-binding reporter construct (ChBD-HuCκ). Images show chitin co-localisation with NOD2 (A and C), TLR9 (A and B) and MR (B and C). Images are representative of two independent experiments, scale bars = 10 µm.

**Figure 6:**
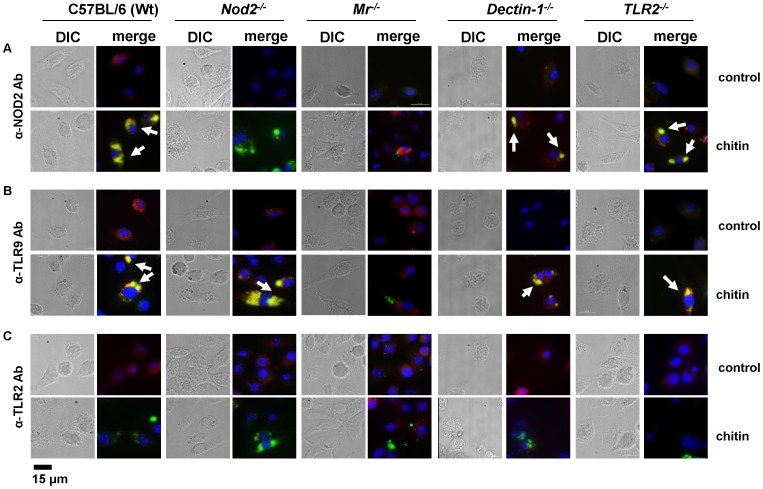
Chitin co-localisation with NOD2 and TLR9 depends on MR. Fluorescence microscopy of mBMMφs from wild type, TLR2-, NOD2-, MR- and dectin-1-deficient mice stimulated with chitin for 1 h. Nuclei are stained with DAPI (blue) and chitin was detected using a chitin-binding reporter construct (ChBD-HuCκ, green). (A) NOD2 protein was detected with anti-mouse NOD2 antibody (red) (B) TLR9 protein was detected with anti-mouse TLR9 antibody (red) and (C) TLR2 protein was detected with anti-mouse TLR2 antibody (red). White arrows indicate co-localisation of chitin with NOD2 and/or TLR9 (yellow). Chitin did not co-localise with TLR2 in all tested cells (C) and no chitin co-localisation with NOD2 or TLR9 was detectable in MR-deficient macrophages (A and B). Images (A to C) are representative of two independent experiments, scale bars = 15 µm.

Microscopy was performed to visualise MR, NOD2 and TLR9 localisation in chitin stimulated macrophages (Fig. 5). Co-localisation of chitin with MR (Fig. 5B) as well as co-localisation of TLR9 (Fig. 5A and B) and NOD2 (Fig. 5A and C) with chitin could be observed after 20 min of incubation. F-actin staining with phalloidin showed phagocytic uptake of chitin (Fig. 5).Fluorescence profiles confirmed overlay of fluorescence signals for MR and chitin (Fig. 5B and C), TLR9 and chitin (Fig. 5A and B) and NOD2 and chitin (Fig. 5A and C). In wild type, *Tlr2^−/−^*- and *dectin-1^−/−^*- macrophages, chitin (green) co-localised with NOD2 (Fig. 6A) and TLR9 (Fig. 6B) as indicated by fluorescence overlay (yellow). Chitin still co-localised with TLR9 in the absence of NOD2 (Fig. 6B). In MR-deficient macrophages, intracellular chitin was detected but no co-localisation with NOD2 and/or TLR9 was observed (Fig. 6A and B). Chitin did not co-localise with TLR2 in wild type macrophages or tested mutants (Fig. 6C). Taken together, these results demonstrate that chitin uptake via MR is required for NOD2- and TLR9-dependent chitin recognition and for the induction of an anti-inflammatory immune response through IL-10 secretion. Blocking chitin uptake mediated by the MR or MR-deficiency shifted the anti-inflammatory response to a pro-inflammatory response through TNF secretion that is Dectin-1 and TLR2-dependent and phagocytosis independent.

### Chitin dampens LPS-induced inflammation *in vivo*

**Figure 7:**
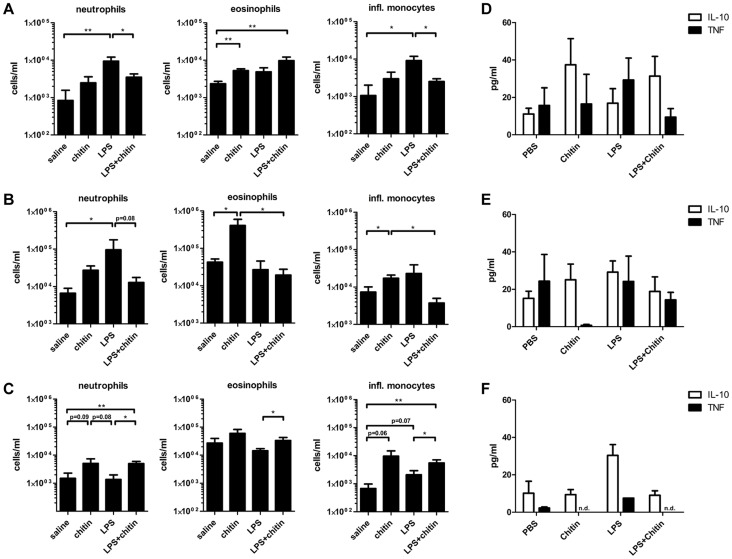
Chitin dampens LPS induced inflammation *in vivo*. C57BL/6 mice were injected intraperitoneal with saline, chitin, LPS or chitin and LPS in combination. Infiltrating immune cells and cytokine production were analysed after 4 h (A and D), 24 h (B and E) and 4 days (C and F). Data are presented as mean values ± SEM, n = 5 mice per group, *p<0.05, **p<0.01.

We showed that fungal chitin particles co-stimulated IL-10 and reduced pro-inflammatory cytokines *in vitro*. Next we investigated the ability of chitin to reduce inflammation *in vivo*. Wild type mice were injected intraperitoneally with saline, chitin (100 µg), LPS (10 µg) or chitin and LPS in combination and infiltrating immune cells and cytokines analysed after 4 h, 24 h and 4 days (Fig. 7 and Supplementary Fig. S4). Injection of chitin, LPS and chitin/LPS significantly increased the number of total infiltrating immune cells in the peritoneal cavity after 4 h compared to saline (Supplementary Fig. S4A). After 24 h, chitin- and LPS-injected mice had increased immune cell infiltrates, whereas chitin/LPS-injected mice had significantly less (Supplementary Fig. S4B). By day 4 no significant differences could be observed between the different groups (Supplementary Fig. S4C).

Inflammatory immune cells, e.g. neutrophils and inflammatory monocytes, were significantly increased in the LPS-injected group after 4 h (Fig. 7A). Chitin injection alone induced only a slight increase of neutrophils and inflammatory monocytes after 4 h, but significantly increased the number of eosinophils after 4 h and 24 h (Fig. 7A and B). After 4 h a combination of chitin and LPS significantly reduced the number of neutrophils and inflammatory monocytes compared to injection of LPS alone (Fig. 7A). Co-injection of chitin with LPS also reduced the number of neutrophils after 24 h (Fig. 7B). Four days after injection, the chitin and LPS/chitin-injected group still had increased numbers of neutrophils, eosinophils and inflammatory monocytes in the peritoneal cavity, whilst the LPS-injected group showed no evidence of inflammation (Fig. 7C).

Cytokine analysis revealed that chitin increased IL-10 levels in the peritoneal cavity after 4 h and 24 h (Fig. 7D and E). LPS-injection increased TNF levels after 4 h (Fig. 7D) and induced equal amounts of IL-10 and TNF secretion after 24 h (Fig. 7E). The combination of chitin together with LPS shifted the cytokine response towards a more pronounced IL-10 response similar to that induced by chitin alone (Fig. 7D and E). In summary, these results show that chitin dampened the inflammatory response to LPS *in vivo*.

### Reduced cell wall chitin in *C. albicans* and inhibition of chitinase activity influences cytokine secretion

**Figure 8:**
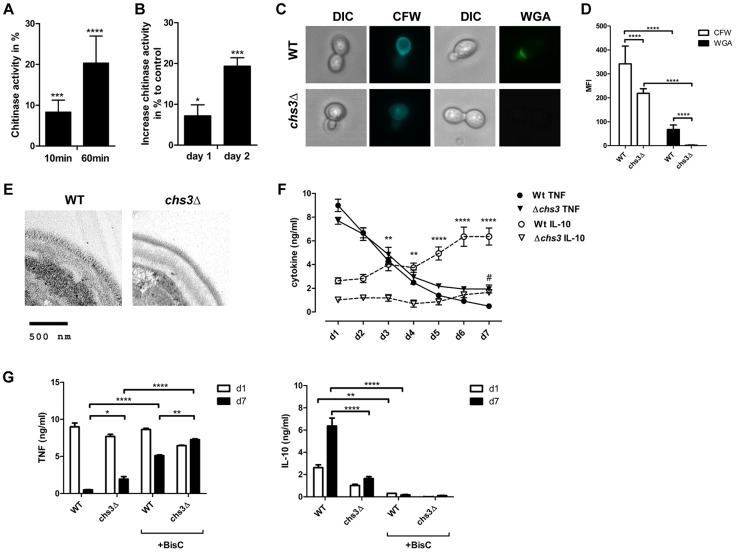
Reduced cell wall chitin effects late phase cytokine response to *C. albicans*. CHIT-1-activity of (A) hPMNs and (B) hMφ's incubated with live *C. albicans* yeast cells, MOI = 0.4. Values represent means ± SEM, n = 4, *p<0.05, ***p<0.001, ****p<0.0001. (C) Heat-treated yeast cells from *C. albicans* wild type and *chs3Δ* mutant were stained for total chitin content with Calcofluor White (CFW) and surface presented chitin with wheat germ agglutinin (WGA) and (D) mean fluorescence intensity (MFI) was analysed. Values are presented as mean ± SEM, n = 30, ****p<0.0001. (E) TEM analysis of heat-treated yeast cells from *C. albicans* wild type and *chs3Δ* mutant. Images shown are representative for all analysed yeast cells. (F and G) hPBMCs were incubated with heat-treated *C. albicans* wild type yeast cells or *C. albicans chs3Δ*, MOI = 0.4 in the presence or absence of the chitinase inhibitor Bisdionin C. TNF and IL-10 secretion was monitored for a period of 7 days. Values represent means ± SEM, n = 4, *p<0.05, **p<0.01, ****p<0.0001.

We also examined whether IL-10-inducing chitin particles were released from fungal cells during infections. Human chitotriosidase (CHIT-1) is expressed constitutively in phagocytic cells, such as neutrophils and macrophages and expression levels increase during monocyte to macrophage differentiation [15]. Stimulation of PRRs (e.g. TLR2, Dectin-1 and NOD2) with their specific ligands has been shown to influence CHIT-1 expression [14], [16] and recombinant human chitotriosidase exhibits antifungal activity against several different fungal species [17]. Nonetheless, it has not been determined whether CHIT-1 secretion increases during fungal infections. We therefore co-incubated human neutrophils (Fig. 8A) and macrophages (Fig. 8B) with *C. albicans* and analysed CHIT-1 activity in the cell supernatants and lysates. The total CHIT-1 activity increased significantly over time for neutrophils (Fig. 8A) and macrophages (Fig. 8B) when stimulated with live *C. albicans* yeast cells in comparison to uninfected cells. These results suggest that IL-10-inducing particles are generated during infection due to the digestion of fungal cell wall chitin by CHIT-1. Next we incubated hPBMCs with heat-treated *C. albicans* yeast cells over a period of 7 days and measured cytokine levels daily. We included heat-treated yeast cells from the *C. albicans* chitin synthase 3 mutant strain (*chs3*Δ) which has a greater than 80% reduction in the total amount of chitin in the cell wall (Fig. 8C and D) [18]. Heat-treatment did not differentially alter the cell wall structure of wild type and *chs3*Δ-mutant as evidenced by transmission electron microscopy (Fig. 8E). Wild type and *chs3*Δ mutant cells induced equal amounts of TNF from day 1 to 4, but as the response to the wild type yeast cells decreased to control levels from day 5 to 7, the amount of TNF in the *chs3*Δ mutant samples remained significantly higher (Fig. 8F). In contrast, in the wild type samples IL-10 levels significantly increased over time, but this did not occur in the chitin-deficient *chs3Δ* mutant samples (Fig. 8F). Finally we investigated the importance of human chitinases in generating IL-10 inducing particles from *C. albicans*. We repeated the experiment described above in the absence or presence of the chitinase inhibitor Bisdionin C (50 µM) (Fig. 8G). The presence of Bisdionin C did not affect the pro-inflammatory TNF response on day 1, but interestingly lead to significantly higher TNF levels on day 7 compared to the samples without Bisdionin C (Fig. 8G). On the other side, inhibition of the chitinase-activity completely abolished the induction of IL-10 in wild type and *chs3*Δ mutant treated samples (Fig. 8G).

**Figure 9:**
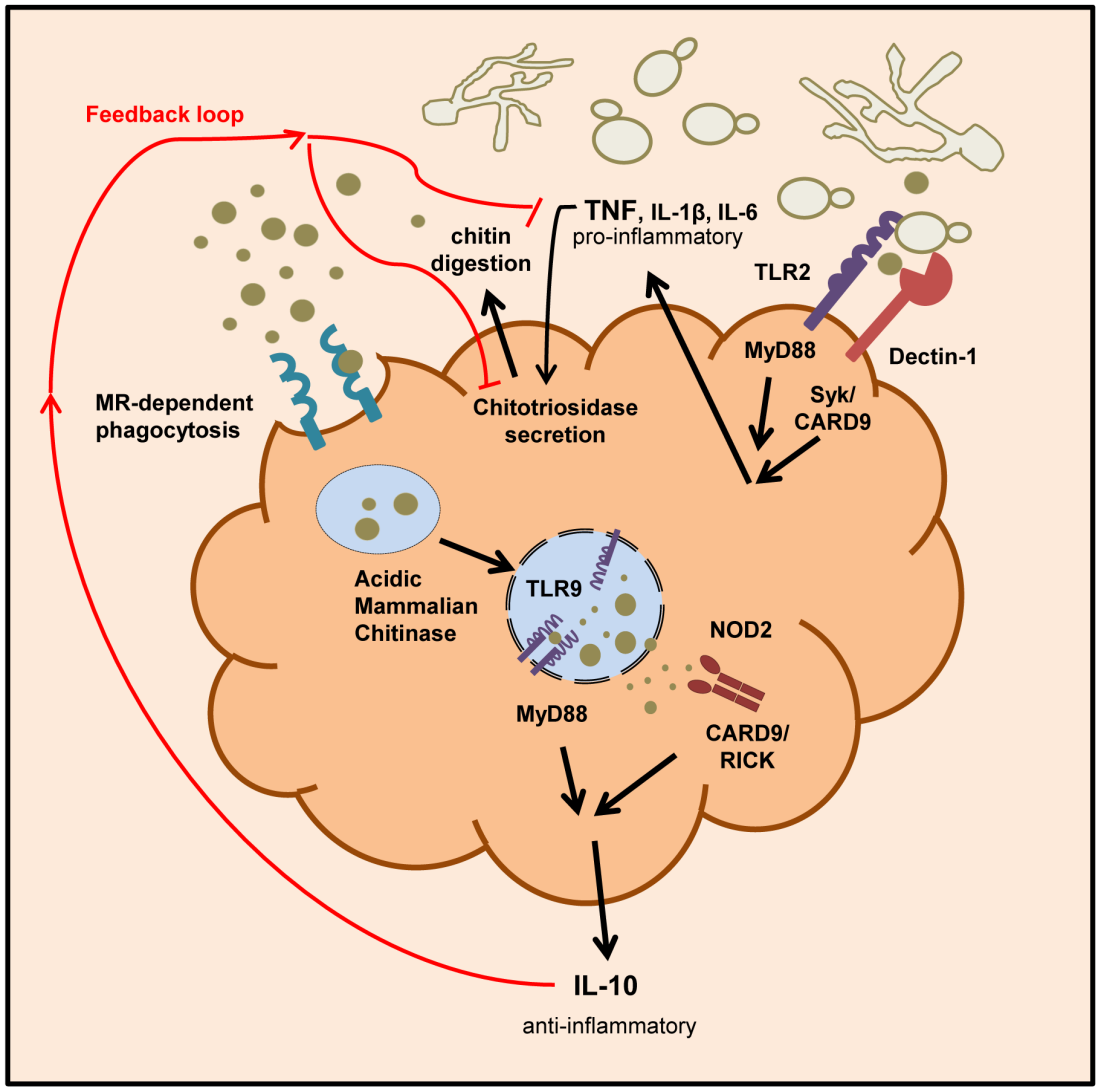
Schematic overview of chitin recognition and involved pathways in negative regulation of inflammation. Innate recognition of fungal cells by PRRs like Dectin-1 and TLR2 leads to the induction of pro-inflammatory cytokines, such as TNF. The pathogen recognition together with the release of pro-inflammatory cytokines induces the secretion of chitinases (e.g. chitotriosidase) from neutrophils and macrophages. Chitin digestion leads to the generation of small sized chitin particles that are taken up by the mannose receptor and induce IL-10 secretion via the TLR9 and NOD2 pathway. The anti-inflammatory cytokine IL-10 dampens the inflammatory response by down-regulating pro-inflammatory cytokine secretion.

These observations indicate that the release of cell wall chitin during the end/late-phase of infection when the pathogen has been defeated by the immune system contribute to the resolution of the immune response by the induction of IL-10 thereby preventing collateral inflammatory-mediated damage (Fig. 9).

## Discussion

Chitin is an abundant molecule in nature and is a component of pathogens, parasites and food products of humans [1], [2], [3], [4]. Chitin exposure and degradation has been linked to a variety of human diseases, such as allergic asthma and Crohn's disease [19]. Therefore, chitin recognition and chitin-triggered immune responses are of broad interest to studies of immunity and inflammation. To date, most immunological studies have mostly used semi-purified commercial sources of chitin derived from shrimp or crab shells [10], making interpretations of the immunological effects of chitin difficult. Here we investigated the immune regulatory ability of ultrapure fungal chitin as a PAMP and describe several classes of immune receptors that are involved in chitin recognition (Fig. 9). We show that fungal chitin acts as a concentration- dependent stimulus of pro- and anti-inflammatory cytokine secretion *in vitro* and *in vivo*. Chitin particles (1–10 µm), resembling a size that is most likely to be found in the natural environment of the host, stimulated mainly IL-10 secretion at lower concentrations. Higher chitin concentrations induced TNF secretion. Lower chitin concentrations were only able to induce TNF secretion when intracellular uptake of chitin was inhibited. In this case, chitin-induced TNF secretion was dependent on TLR2 and dectin-1 recognition as blocking chitin uptake abolished TNF induction in TLR2- and dectin-1-deficient macrophages. Our findings support and explain recent observations reporting that chitin acts as a size-dependent stimulus of IL-10 and TNF, mediated through MR, TLR2 and dectin-1 signalling pathways [13], but also help explain observed variations in chitin-induced immune responses related to chitin particle size and chitin concentration [20].

Whilst the inhibition of chitin uptake led to an increase chitin-induced TNF secretion, IL-10 induction was markedly increased by liposomal transfection, indicating that intracellular recognition was responsible for the anti-inflammatory properties of chitin. By screening mice that were deficient for various PRRs, we showed that NOD2 and TLR9 were required for chitin recognition, IL-10 induction and TNF suppression. Interaction with these two intracellular receptors required chitin to be first recognised by the MR before delivered to the inside of the cell since *Mr−/−* macrophages failed to induce IL-10, even when chitin was delivered using DOTAP as a liposomal transfecting agent. TLR9 recognises unmethylated CpG motifs in bacterial DNA, and has also been shown to recognise fungal DNA [21]. However, recent studies implicate TLR9 in fungal recognition independent of fungal viability or DNA availability, and TLR9 recruitment to fungus-loaded phagosomes has been shown to depend on the inner fungal cell wall layer rather than the outer wall mannoproteins [22]. In addition, siRNA knock-down of TLR9 expression in hPBMCs demonstrated that TLR9 signalling suppresses pro-inflammatory cytokine secretion (e.g. TNF and IL-6) and antimicrobial activity [23]. Interestingly, a protective role for TLR9 in acute allergic airway diseases driven by *Aspergillus spp* or house mite containing dust has been implied, since CpG administration inhibits allergic Th2 cytokine production [24], [25]. This effect might be mediated though competitive binding of CpG and chitin to TLR9, as CpG was the only PAMP used in our experiments, which did not show a synergistic effect on IL-10 secretion together with chitin. TLR2, dectin-1 and TLR9 have also been shown to be required for *Aspergillus fumigatus* strains to exhibit paradoxical growth upon exposure to caspofungin inside the host [26]. Paradoxical growth during echinocandin treatment has been linked to the ability of the fungi to upregulate chitin synthesis to restore the cell wall integrity after echinocandin-mediated cell wall damage [7], [27]. We showed previously that *C. albican*s cells with high chitin levels in their cell wall survive exposure to caspofungin in a mouse infection model [8], and that mice inoculated with high-chitin cells do not exhibit the inflammation-based pathology of the kidney that is characteristic of infection with *C. albicans* cells with normal chitin levels. Subsequently, mice with high kidney burdens of chitin-rich yeast cells survived infection [8]. Therefore, the immunopathology of fungal infections and the immune response of patients treated with echinocandins may be influenced by the recognition of cell wall chitin and the consequential dampening or exacerbation of the antifungal inflammatory response.

TLR9 SNPs have been linked with a higher susceptibility to CD [28] - a disease which is also associated with mutations in the NOD2 gene. Moreover, recent studies suggest that the normal immune interactions between TLR9 and NOD2 are lost in CD patients [28], [29]. NOD2 is a cytosolic innate immune receptor that mediates pro-inflammatory and antibacterial responses through recognition of bacterial cell wall components. The underlying mechanism governing how NOD2 variants lead to CD development is unknown, but current hypotheses suggest that the impaired function of NOD2 leads to deficiencies in the epithelial-barrier function, leading to increased bacterial invasion and inflammation of the intestine [30]. *C. albicans* colonises all segments of the digestive tract without any known benefit for the host, but commensal colonisers are also the source of fungal cells that invade the mucosa, leading to life-threatening systemic infections in immunocompromised individuals [31]. Recent investigations described high serum levels of antibodies in 50–60% of CD patients, that recognise major epitopes of the fungal cell wall, including mannan, β-glucan and chitin [32]. These antibodies are produced during *C. albicans* infection and normally subside after antifungal therapy; however serum levels remain high in CD patients [32]. Interestingly, it has been demonstrated that healthy relatives of CD patients are more frequently colonised by *C. albicans* in the gut compared to healthy control families [33]. Moreover, analysis of the gut fungal microbiota in healthy and CD patients revealed that the fungal diversity is significantly elevated in inflamed mucosa compared to healthy controls, thereby showing an expansion of pathogenic species like *Candida* spp [34]. Therefore the fungal gut microflora may be an important immunomodulator of CD.

The Th1/Th17 response is known to be protective against mucosal *Candida* infections [35], however Th17 responses have also been implicated in the pathology of CD, allergy and asthma [36]. Elevated IL-17 levels and Th17 cells are found in the intestinal mucosa of CD patients [37] and IL-10-treatment of mice with established colitis suppressed Th17 and Th17/Th1 cell development [38]. IL-10 has an important role in regulating gut immunity and intestinal homeostasis, and CD-associated NOD2 mutations have been shown to inhibit IL-10 expression [39]. Two recent studies investigated the modulation of intestinal inflammation by yeast cell wall extracts (*C. albicans* and *S. cerevisiae*) and chitin micro particles (1–10 µm), both demonstrated increased IL-10 levels and stimulation of IL-10 producing cells in the colon with consequent improvement of inflammation [40], [41].

Low concentrations of small chitin particles are likely to be released by commensal fungi in the gut and on other mucosal surfaces, as well as from the cell walls of fungi that have been killed by the action of the immune system. The release of such chitin particles and chitin oligosaccharides has the potential to induce IL-10 secretion, via NOD2 and TLR9 signalling, promoting the attenuation of inflammatory-mediated diseases and consequent immune homeostasis [42]. High concentrations of chitin particles generated during pathogen invasion and infection may promote inflammation through dectin-1 and TLR2 signalling. However, chitin is also able to promote Th2-associated inflammation which is central to the pathogenesis of allergy and asthma [43], [44]. Our data therefore suggest that the two signature polysaccharides in the fungal cell wall, β-1,3 glucan and chitin, induce markedly different responses in the sentinel cells of the innate immune system and explain why chitin and chitin recognition pathways are implicated in the immunology of asthma, CD and allergies.

## Materials and Methods

### Chitin purification and analysis

*C. albicans* strain NGY152 [45] was used in this study as a main source of chitin. All other strains used in this study are listed in Supplementary Table S1. Chitin was isolated and purified as described previously [46] with minor adaptations. Briefly, cells were grown in YPD broth (1% (w/v) yeast extract, 2% (w/v) mycological peptone, 2% (w/v) glucose) at 30°C with shaking at 200 rpm overnight. Cells were harvested by centrifugation, washed three times with deionised water, resuspended in 5% (w/v) KOH and boiled at 100°C for 30 min. This procedure was repeated twice before cells were resuspended in 40% H_2_O_2_/glacial acetic acid solution (1∶1) and boiled at 100°C for 45 min. Chitin was collected by low speed centrifugation and washed several times with deionised water before being resuspended in phosphate buffered saline (PBS) and stored at 4°C.

To analyse the quantity and purity of the chitin preparations, samples were hydrolysed with 13 M (99% (w/v)) trifluoroacetic acid (TFAA) at 100°C for 4 h for purity analysis or 6 M HCl at 100°C overnight for chitin yield measurements. Acid was evaporated at 65°C–70°C, the debris washed twice with deionised water by evaporation and finally resuspended in deionised water. Samples were analysed together with carbohydrate standards by high-performance anion-exchange chromatography with pulsed amperometric detection (HPAEC-PAD) in a carbohydrate analyzer system from Dionex (Surrey, UK) as described previously [47]. Prior to experiments, chitin samples were tested for endotoxin contamination using the limulus amebocyte lysate (LAL) assay (QCL-1000; Lonza) and the amount detected was less than 0.3 EU/ml (<30 pg/ml). The degree of chitin acetylation/deacetylation was determined by Cibacon Brilliant Red 3B-A dye binding [48]. Possible bacterial and fungal contamination were excluded by incubating 20 µl of each chitin sample in YPD broth (fungi) or LB broth (1% (w/v) tryptone, 0.5% (w/v) yeast extract, 0.5% (w/v) NaCl) (bacteria) for 24 h to 4 h at 37°C. Chitin particle size was determined by flow cytometry using 1 and 10 µm latex beads as size standards (Sigma-Aldrich) as described elsewhere [49].

### Microbial ligands and chemicals

All chemicals used in the study were of cell culture grade and endotoxin-free. Pattern recognition receptor ligands used in this study were purchased from InvivoGen (LPS, Pam_3_CSK_4_, CpG ODN 2395, zymosan, MDP and flagellin) and curdlan, laminarin and *S.c.* mannan from Sigma-Aldrich. All samples were prepared in endotoxin-free water. The endocytosis inhibitor Cyt D was purchased from Sigma-Aldrich, the transfection reagent DOTAP from Roche and DNase I from Invitrogen. The chitinase inhibitor Bisdionin C was a kind gift from I. Eggleston (Bath).

### Ethics statement, animals and receptor-deficient mice

All animal experiments were approved by ethical committees of respective institutes, and conducted according to local guidelines and regulations. For all experiments age- and sex-matched KO mice and co-housed wild type animals between 6–12 weeks of age were used.

C57BL/6 (wild type), *Tlr2^−/−^*, *Nod2^−/−^*, *Myd88^−/−^*, *Card9^−/−^* and *Rick^−/−^* mice were housed in the SJCRH animal resource center, which is a specific pathogen free (SPF) and AAALAC accredited facility. Experiments were conducted under protocols approved by the St. Jude Children's Research Hospital Committee on Use and Care of Animals (Protocol #482) and were performed in accordance with institutional policies, AAALAC guidelines, the AVMA Guidelines on Euthanasia NIH regulations (Guide for the Care and Use of Laboratory Animals), and the United States Animal Welfare Act (1966).

C57BL/6, *Tlr2^−/−^*, *Tlr4^−/−^*, *Tlr2/4^−/−^*, *Nod2^−/−^* and *Tlr9^−/−^* mice were bred under specific pathogen-free conditions in the animal facility of the University of Tübingen according to European guidelines (FELASA) and to the guidelines for the care and use of laboratory animals of the German Animal Protection Law. Protocols were approved by the board institution animal facility of the University of Tübingen and the local authorities Regierungspräsidium Tübingen with the permit numbers §4 Abs. 3 Az v. 01.07.09 according to German Animal Protection Law. *Tlr2^−/−^* mice were a kind gift from C. Kirschning (Technical University Munich), *Tlr4^−/−^* mice were kindly provided by Dr. S. Akira (Osaka University). *Tlr2/4^−/−^* mice were generated by mating TLR2-deficient mice with TLR4-deficient mice.

C57BL/6 (wild type), *Mr^−/−^*, 129/Sve (wild type) and *Clec7a^−/−^* (*dectin-1^−/−^*) mice were bred and housed under pathogen-free conditions in the animal facility at the University of Aberdeen. Experimentation involving animals was carried out under a UK Home Office Project Licence granted to Dr Donna MacCallum (PPL 60/4135) and Prof Gordon Brown (PPL60/4007), and all work was approved by the UK Home Office and the University of Aberdeen Ethical Review Committee, and conforms to European Union directive 2010/63/EU. *Mr^−/−^* mice were a kind gift from S. Gordon (Oxford).

### *In vivo* stimulation and analysis

Mice were injected intraperitoneally with indicated concentrations of chitin and LPS in 200 µl sterile saline. Mice were sacrificed at the indicated time points and peritoneal inflammatory cells were harvested in PBS containing 5 mM EDTA. Cells were stained with CD11b-PECy7, CD11c-PerCPCy5.5, 7/4-FITC, SiglecF-PE, Ly6G-APC and F4/80-AF700 (BD Biosciences) to distinguish neutrophils (F4/80^neg^, 7/4^pos^, Ly6G^high^), eosinophils (F4/80^neg^, 7/4^neg^, Ly6G^pos^, SiglecF^pos^), inflammatory monocytes (F4/80^pos^, 7/4^pos^, Ly6G^neg^) and macrophages (F4/80^pos^, 7/4^neg^, Ly6G^neg^). Data was acquired on FACS LSRII and analysed using FlowJo.

### Isolation, differentiation and cytokine production by mouse bone-marrow derived macrophages

Bone marrow-derived macrophages (mBMMφs) were prepared as described previously [50]. Briefly, bone marrow was isolated from femurs of 6–12 week-old mice and cultured in IMDM (Gibco) containing 10% heat-inactivated fetal bovine serum (FBS), 20% L929 cell-conditioned medium, 1% MEM non-essential amino acids (NEAA (100×), Gibco), 100 U/ml penicillin and 100 mg/ml streptomycin at 37°C in a humidified atmosphere containing 5% CO_2_. After 5–7 days cells were collected and plated in 96-well plates at a density of 1×10^5^ cells/well in IMDM containing 10% heat-inactivated FBS, 1% NEAA and antibiotics. Macrophages were cultured overnight and primed with 1 µg/ml LPS for 4 h prior to main experiments. Supernatants and cells were then separated and stored at −20°C until cytokine assays were performed. Cyt D treated cells were lysed with 0.1% Triton-X 100 in media at room temperature for 60 min. Cell lysates were stored at −20°C until cytokine measurements were performed.

### Isolation of human peripheral blood mononuclear cells, neutrophils and monocyte to macrophage differentiation

Human polymorphonuclear (hPMNs) and peripheral blood mononuclear cells (hPBMCs) were isolated from EDTA-blood samples, freshly taken from healthy donors according to the local guidelines and regulations, approved by the College Ethics Review Board of the University of Aberdeen (CERB/2012/11/676). Polymorphonuclear and mononuclear cell fractions were obtained by density centrifugation using Histopaque-1119 and -1077 (Sigma-Aldrich) according to the manufactures instructions. hPBMCs were washed twice with PBS and suspended in RPMI 1640 (Dutch modification) supplemented with 2 mM L-glutamine, 1 mM sodium pyruvate and 100 µg/ml gentamicin for stimulation experiments and transferred into a 96-well suspension culture plate at a density of 5×10^5^ cells/well. After experiments supernatants were collected and stored at −20°C until cytokine assays were performed.

To obtain human macrophages (hMφs), mononuclear cells were suspended in RPMI 1640 (Dutch modification) supplemented with 10% heat-inactivated FBS, 2 mM L-glutamine, 1 mM sodium pyruvate, 1% MEM non-essential amino acids (NEAA (100×), Gibco), 100 U/ml penicillin, and 100 mg/ml streptomycin and 50 ng/ml recombinant hGM-CSF (Gibco), seeded into cell culture flasks and incubated at 37°C in a humidified atmosphere containing 5% CO_2_. After 5–7 days cells were collected and seeded into 12-well plates at a density of 5×10^5^ cells/well and cultured overnight before stimulation. Supernatants and cells were separated after experiments and stored at −20°C until further assays were performed.

hPMNs were washed twice with PBS after isolation step and remaining red blood cells were lysed using hypotonic salt solutions. hPMNs were suspended in RPMI 1640 supplemented with 0.5% heat-inactivated FBS. hPMNs (2×10^6^ cells) were stimulated with live *C. albicans* yeast cells (2×10^6^ cells, MOI = 1) for 10 and 60 min. hPMNs were separated from the supernatant by centrifugation and cells were lysed in 0.1% (v/v) TritonX-100 in PBS. Cell lysates and supernatants were stored at −20°C until further analysis.

### Stimulation with heat-treated yeast cells and chitin determination

*C. albicans* wild type and *chs3*Δ-null mutant strains were grown overnight in YPD broth at 30°C with shaking at 200 rpm. Cells were diluted to an OD_600_ of 0.2 in fresh YPD broth and cultured at 30°C until cells reached mid-exponential growth phase (OD_600_ 0.6–0.8). Yeast cells were harvested and washed twice with PBS before they were heat killed by incubation at 65°C for 2 h. hPBMCs (5×10^5^ cells/well) were incubated for 7 days with 2×10^5^ yeast cells (MOI = 0.4). Aliquots of supernatant were collected each day and stored at −20°C until cytokine assays were performed.

Surface presentation of chitin was analysed by staining of heat-treated yeast cells with FITC conjugated wheat germ agglutinin (WGA-FITC, 100 µg/ml) in the dark for 30 min and total cell wall chitin was stained with Calcofluor White (CFW, 10 µg/ml).

### Chitotriosidase-activity assay

Chitotriosidase-activity in the supernatants and lysates of stimulated cells was analysed as described elsewhere [51]. Briefly, chitinase-activity was analysed by mixing 10 µl sample with 100 µl substrate (20 µM 4-methylumbelliferyl β-d-N,N′-diacetylchitotrioside hydrate (4-[MU]-(GlcNAc)_3_, Sigma-Aldrich) in McIlvain buffer (0.1 M citric acid, 0.2 M sodium phosphate, pH 5.2) and incubated for 20 min at 37°C in the dark. The reaction was stopped by adding 200 µl 0.3 M glycine-NaOH buffer, pH 10.5 and converted substrate was measured fluorometrically (Ex.360/Em.450 nm). A serial dilution of 4-[MU] was used as standard.

### Immunofluorescence and high-pressure, freeze-substitution transmission electron microscopy

Bone marrow-derived macrophages were plated into chamber-slides (BD Falcon) at a density of 1×10^5^ cells/chamber and grown overnight prior to stimulation. After stimulation, cells were fixed with 4% (w/v) paraformaldehyde in PBS for 15 min at room temperature or at 4°C overnight and permeabilised with 0.5% (v/v) Triton X-100 in PBS for 5–10 min. Non-specific binding was blocked with 5% goat serum in PBS for 30 min, before samples were stained using anti-NOD2 (clone H-300), anti-TLR9 (clone H-100), anti-TLR2 (clone H-175) (Santa Cruz Biotechnology), anti-TLR9 (clone1E8) (Sigma-Aldrich) or anti-CD206 (cloneMR5D3) (AbD Serotec) together with the chitin-binding reporter construct (ChBD-HuCκ) that contained the chitin binding domain (ChBD) from *Bacillus circulans* chitinase A1 fused to the human kappa light chain (HuCκ) and a 6×His tag for purification (Lenardon M.D., unpublished), followed by detection with the fluorochrome-coupled goat anti-mouse-TRITC (Sigma-Aldrich), goat anti-mouse-AF647 (Molecular Probes), goat anti-rabbit-TRITC (Sigma-Aldrich), goat anti-rat-TRITC (AbD Serotec), goat anti-rat-AF647 (Molecular Probes) or goat anti-human-κ light chain-FITC secondary antibody (Sigma-Aldrich). Slides were mounted with mounting medium containing DAPI (Vector Laboratories).

For structural cell wall analysis *C. albicans* wild type and *chs3Δ*-mutant were grown in YPD at 30°C overnight, diluted into fresh media and harvested in mid-exponential growth phase. Cells were heat-killed at 65°C for 2 h before processed for transmission electron microscopy as described elsewhere [52].

### Statistical analyses

All experiments were performed at least 3 times and revealed comparable results. Results are presented as mean ± SEM. Statistical significance was determined using One-way ANOVA or Two-way ANOVA followed by post-hoc analysis including Tukey method. A *P* value of less than 0.05 was considered significant.

## Supporting Information

Figure S1 **Synergistic effect of chitin on IL-10 secretion.** Co-incubation of hPBMCs with either LPS (10 µg/ml), zymosan (10 µg/ml), curdlan (100 µg/ml), Pam_3_CSK_4_ (1 µg/ml), flagellin (100 ng/ml), CpG ODN (1 µM) or *C. albicans* cell wall proteins (CWPs, 100 µg/ml) and chitin (10 µg/ml) for 24 h, values are means ± SEM, n = 6, *p<0.05.
(TIF)

Figure S2 **Chitin induced TNF secretion in MR-deficient cells is Dectin-1-dependent.** mBMMφs from dectin-1- and MR –deficient mice were incubated with *S. c.* mannan or laminarin 1 h prior stimulation with chitin, n = 4. All data are presented as mean values ± SEM, *p<0.05.
(TIF)

Figure S3 **Effect of DNase I-treatment of chitin induced IL-10 secretion.** Chitin samples were pre-incubated with DNase I for 1 h before added to mBMMφs from wild type mice (C57BL/6) for 24 h. Cytokine secretion was determined by ELISA, and values represent means ± SEM, n = 4, ****p<0.0001.
(TIF)

Figure S4 **Chitin dampens LPS induced inflammation in vivo.** C57BL/6 mice were injected intraperitoneal with saline, chitin (100 µg), LPS (10 µg) or chitin and LPS in combination. Infiltrating immune cells and cytokine production were analysed after 4 h (A and D), 24 h (B and E) and 4 days (C and F). Data are presented as mean values ± SEM, n = 5 mice per group, *p<0.05, **p<0.01, ****p<0.0001.
(TIF)

Table S1 **Fungal strains used in the study.**
(DOC)
