# Supplementary figures and images for: Fungal Chitin Dampens Inflammation through IL-10 Induction Mediated by NOD2 and TLR9 Activation
Source: PLoS Pathog. 2014 Apr 10;10(4):e1004050. doi: 10.1371/journal.ppat.1004050 (PMC3983064; doi:10.1371/journal.ppat.1004050)

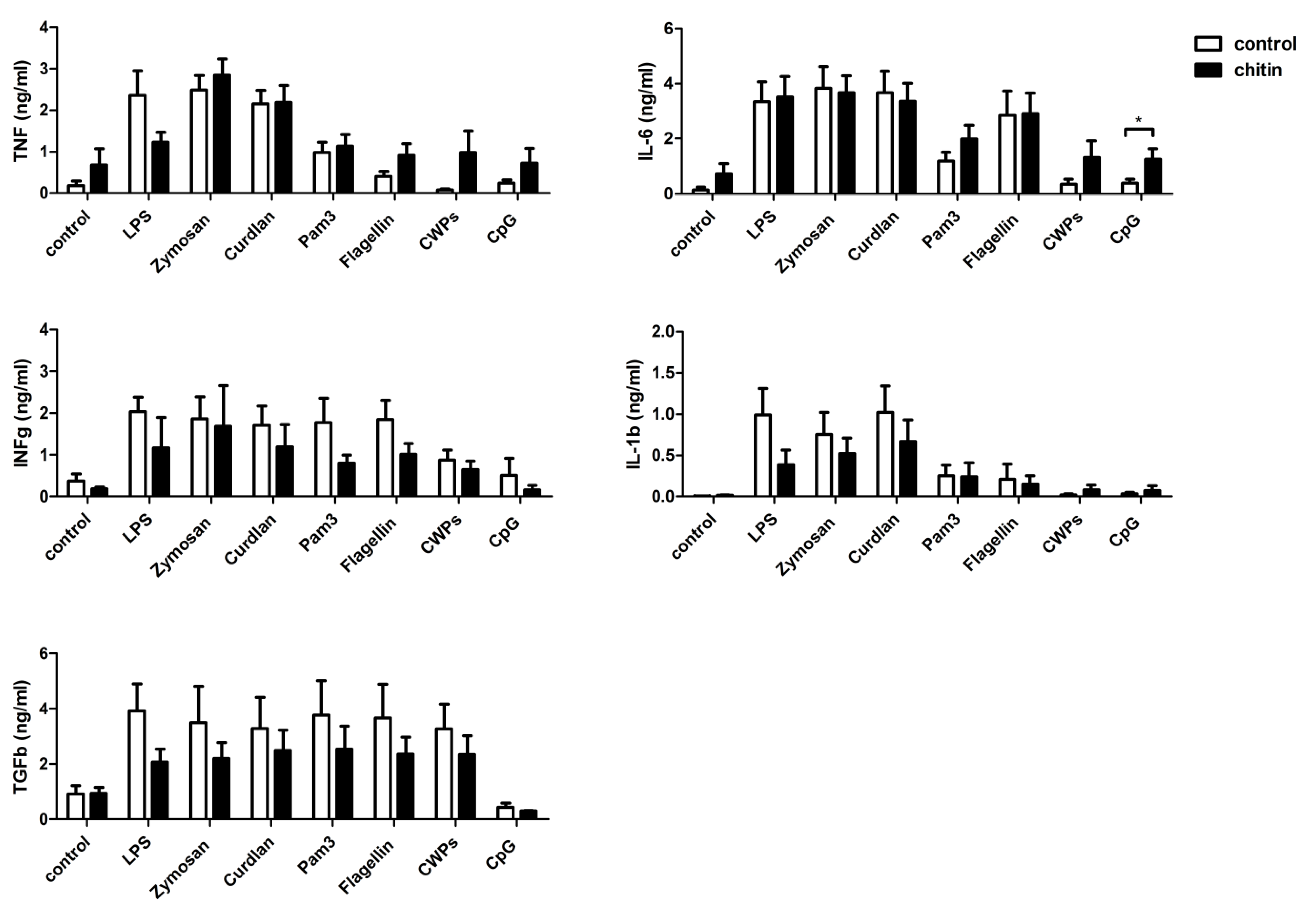

Supplement: Figure S1 — Synergistic effect of chitin on IL-10 secretion. Co-incubation of hPBMCs with either LPS (10 µg/ml), zymosan (10 µg/ml), curdlan (100 µg/ml), Pam3CSK4 (1 µg/ml), flagellin (100 ng/ml), CpG ODN (1 µM) or C. albicans cell wall proteins (CWPs, 100 µg/ml) and chitin (10 µg/ml) for 24 h, values are means ± SEM, n = 6, *p<0.05. (TIF) [file ppat.1004050.s001.tif]

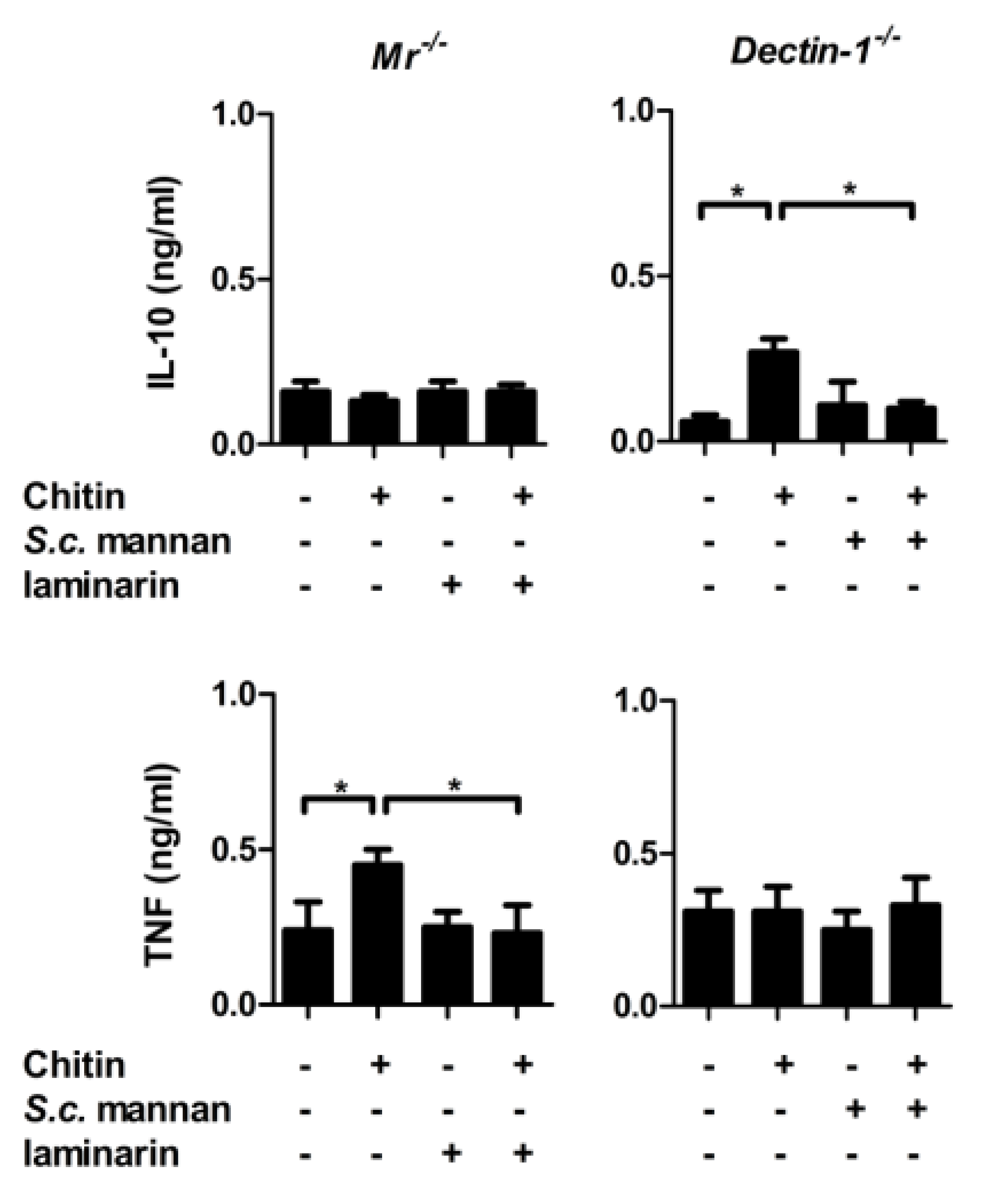

Supplement: Figure S2 — Chitin induced TNF secretion in MR-deficient cells is Dectin-1-dependent. mBMMφs from dectin-1- and MR –deficient mice were incubated with S. c. mannan or laminarin 1 h prior stimulation with chitin, n = 4. All data are presented as mean values ± SEM, *p<0.05. (TIF) [file ppat.1004050.s002.tif]

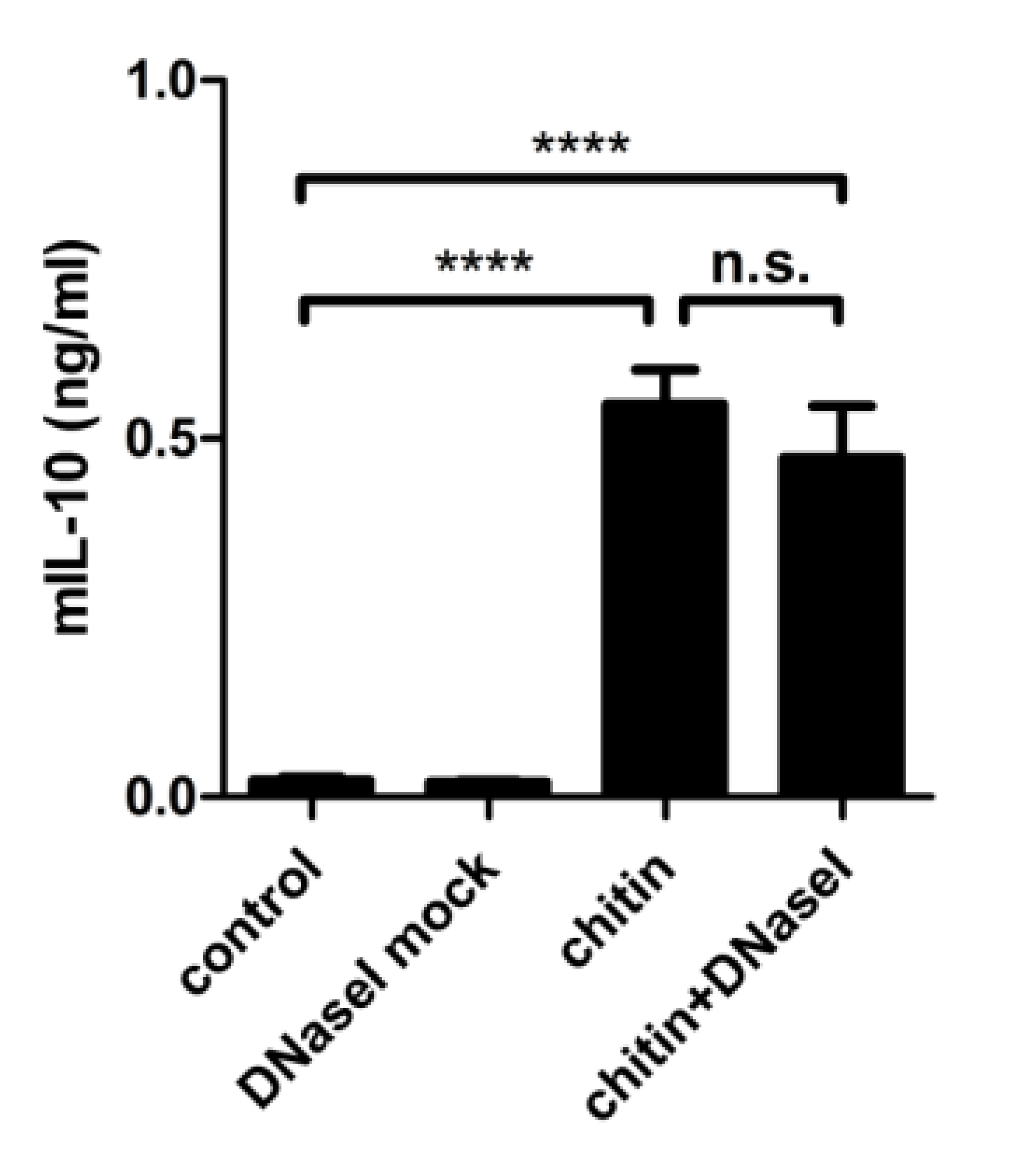

Supplement: Figure S3 — Effect of DNase I-treatment of chitin induced IL-10 secretion. Chitin samples were pre-incubated with DNase I for 1 h before added to mBMMφs from wild type mice (C57BL/6) for 24 h. Cytokine secretion was determined by ELISA, and values represent means ± SEM, n = 4, ****p<0.0001. (TIF) [file ppat.1004050.s003.tif]

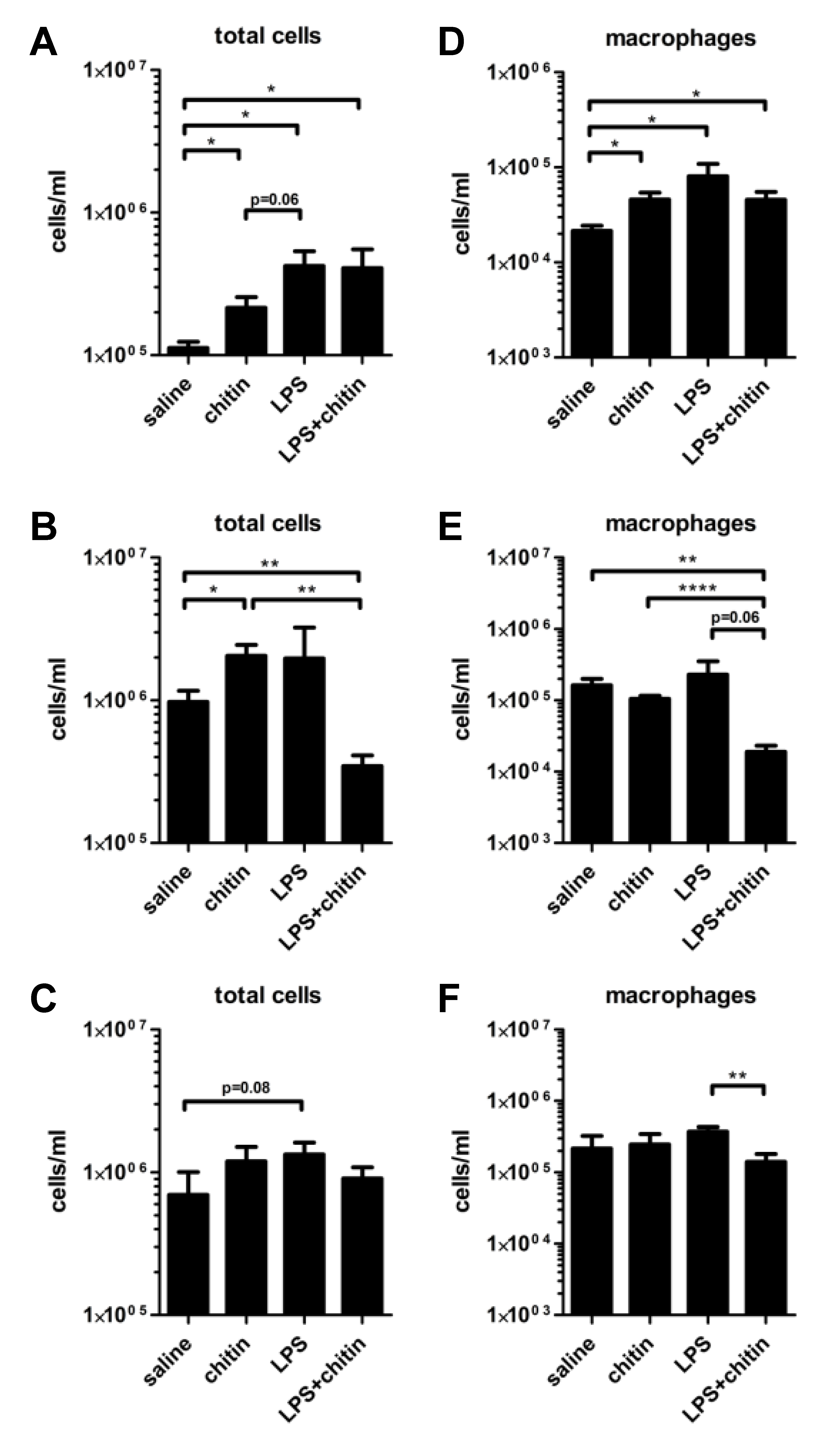

Supplement: Figure S4 — Chitin dampens LPS induced inflammation in vivo. C57BL/6 mice were injected intraperitoneal with saline, chitin (100 µg), LPS (10 µg) or chitin and LPS in combination. Infiltrating immune cells and cytokine production were analysed after 4 h (A and D), 24 h (B and E) and 4 days (C and F). Data are presented as mean values ± SEM, n = 5 mice per group, *p<0.05, **p<0.01, ****p<0.0001. (TIF) [file ppat.1004050.s004.tif]
