# Supplementary material for: Fungal Chitin Dampens Inflammation through IL-10 Induction Mediated by NOD2 and TLR9 Activation
Source: PLoS Pathog. 2014 Apr 10;10(4):e1004050. doi: 10.1371/journal.ppat.1004050 (PMC3983064; doi:10.1371/journal.ppat.1004050)
Supplement: Table S1 — Fungal strains used in the study. (DOC) [file ppat.1004050.s005.doc]

**Supplementary Information**

**Table S1 Fungal strains used in the study**

| **Species** | **Strain name** | **Reference** |
| --- | --- | --- |
| *C. albicans* wild type | NGY152 | [1] |
| *C. albicans ∆chs3* | myco3 | [2] |
| *A. fumigatus* | 237 | [3] |
| *S. cerevisiae* | BY4741 | [4] |
| *M. circinelloides* | CBS277.49 | [5] |
| *C. neoformans* | H99 | [6] |

**Supplementary references**

1. Brand A, MacCallum DM, Brown AJ, Gow NA, Odds FC (2004) Ectopic expression of URA3 can influence the virulence phenotypes and proteome of *Candida albicans* but can be overcome by targeted reintegration of URA3 at the RPS10 locus. Eukaryot Cell 3: 900-909.

2. Bulawa CE, Miller DW, Henry LK, Becker JM (1995) Attenuated virulence of chitin-deficient mutants of *Candida albicans*. Proc Natl Acad Sci U S A 92: 10570-10574.

3. Mellado E, Dubreucq G, Mol P, Sarfati J, Paris S, et al. (2003) Cell wall biogenesis in a double chitin synthase mutant (chsG-/chsE-) of *Aspergillus fumigatus*. Fungal Genet Biol 38: 98-109.

4. Brachmann CB, Davies A, Cost GJ, Caputo E, Li J, et al. (1998) Designer deletion strains derived from Saccharomyces cerevisiae S288C: a useful set of strains and plasmids for PCR-mediated gene disruption and other applications. Yeast 14: 115-132.

5. Li CH, Cervantes M, Springer DJ, Boekhout T, Ruiz-Vazquez RM, et al. (2011) Sporangiospore size dimorphism is linked to virulence of *Mucor circinelloides*. PLoS Pathog 7: e1002086.

6. Perfect JR, Lang SD, Durack DT (1980) Chronic cryptococcal meningitis: a new experimental model in rabbits. Am J Pathol 101: 177-194.
